# Hope as a predictor of physical activity behavior in middle-aged and older adults with musculoskeletal pain

**DOI:** 10.3389/fpsyg.2025.1572256

**Published:** 2025-04-29

**Authors:** Renee Kessler, Monica M. Teegardin, Anthony S. Kaleth, Kelly M. Naugle

**Affiliations:** Indiana University Indianapolis, School of Health and Human Sciences, Indianapolis, IN, United States

**Keywords:** trait hope, pathways, physical activity, musculoskeletal pain, older adults

## Abstract

**Background:**

Musculoskeletal pain is a barrier to physical activity, enhancing functional decline in older adults. Thus, identifying psychological factors that promote physical activity in older adults with musculoskeletal pain is warranted. Prior research shows that the psychological construct of hope predicts the frequency of exercise in healthy younger adults. However, the impact of hope on physical activity behavior in an older population with clinical pain is unknown. This observational study was designed to determine whether hope predicted self-reported and objective physical activity levels in older adults with musculoskeletal pain.

**Methods:**

Fifty-two middle-aged to older adults (age range 55–85 years; 67% female) completed all assessments. Participants completed questionnaires to assess hope (Adult Hope Scale), self-reported physical activity (Physical Activity Scale for the Elderly), bodily pain (SF-36), kinesiophobia (Tampa Scale of Kinesiophobia), and pain catastrophizing (Pain Catastrophizing Scale). Participants also wore accelerometers on the hip for one week to objectively measure physical activity levels. Correlations were conducted to determine relationships between variables. Hierarchical regressions were conducted to determine whether hope predicted self-reported and objective physical activity levels after controlling for relevant demographics, pain, and other psychological variables.

**Results:**

After controlling for bodily pain, hope significantly predicted self-reported physical activity and was associated with greater physical activity levels. Bodily pain, but not hope, significantly predicted average daily steps derived from the accelerometer. Decreased bodily pain was associated with more daily steps.

**Conclusion:**

These findings suggest that trait hope could be a key psychological predictor of self-reported physical activity in older adults with musculoskeletal pain. Clarifying the role of hope in the physical activity behavior of older adults could present a novel target for intervention.

## 1 Introduction

Chronic musculoskeletal pain in older adults is a major public health concern and a leading cause of disability, affecting 1.71 billion individuals globally and accounting for 300 billion dollars in healthcare costs annually (Welsh et al., 2020). Musculoskeletal pain is the most common type of pain in older adults and has biological, psychological, and social implications, posing a threat to healthy aging (Blyth and Noguchi, 2017). Research suggests that physical activity (PA) is critical in the management of musculoskeletal conditions. Yet, pain and pain-related fear are frequent barriers to PA engagement for this population, contributing to worsening health outcomes. Thus, identifying biopsychosocial factors that promote PA in older adults with musculoskeletal pain is warranted.

Accumulating evidence suggests the psychological construct of hope impacts behavior in a variety of contexts (Anderson and Feldman, 2020; Curry et al., 1997; Snyder et al., 1991). Snyder (Yarcheski et al., 2004) defines hope as an individual’s ability to strategize pathways and cultivate agency to think in alignment with goals. Pathways represent the mental process of planning different ways to achieve goal attainment. Agency is conceptualized as an individual’s belief in their ability to act according to the pathways, which contributes to their level of motivation to think in goal-directed terms (Yarcheski et al., 2004). Individuals who foster more hope tend to cultivate more positive emotions, allowing the cognitive capacity to overcome obstacles toward goals (Anderson and Feldman, 2020). Individuals with low dispositional hope have a decreased capacity for developing pathways and cultivating agency, which influences subsequent emotions and decreases their motivation to participate in goal-directed behavior (Anderson and Feldman, 2020).

Research suggests that hope could be a predictor of positive health behavior, contributing to lower self-reported pain intensity and interference in patients with chronic pain (Yarcheski et al., 2004; Shanahan et al., 2021). Another study examining the influence of hopeful versus fear-based messaging during COVID-19 revealed that individuals who received hopeful messages were more likely to adopt preventative behaviors, such as vaccination, to maintain optimal health amidst the health crisis (Lin et al., 2024). Anderson and Feldman (2020) examined the role of general and goal-specific hope, self-efficacy, and optimism on exercise behavior in a nonclinical younger adult sample. The results revealed that goal-specific hope, particularly agency, was independently associated with greater exercise frequency, even after controlling for other psychological variables (Anderson and Feldman, 2020). Similarly, Goubert et al. (2004) revealed that higher trait hope (i.e., hope measured without reference to a particular goal), particularly agency, longitudinally predicted perceived exercise goal attainment and greater vigorous PA in college students. It is important to note that PA is used to describe any form of bodily movement that results in energy expenditure, while exercise is a structured form of PA designed to improve or maintain physical fitness. The role of hope facilitating PA amongst populations that are likely to face greater barriers to PA, such as those with clinical pain, has not been explored. Based on Hope theory and the aforementioned research, it is hypothesized that pathways, rather than agency, will predict PA behavior in older adults with musculoskeletal pain.

The purpose of this observational study was to determine whether hope predicted self-reported and objective PA behavior in middle-aged to older adults with musculoskeletal pain. We assessed hope, clinical pain, kinesiophobia, pain catastrophizing, and both subjective (self-report) and objective (accelerometery) PA levels in middle-aged and older adults with musculoskeletal pain. We hypothesized that hope, particularly agency, would predict PA levels, even after controlling for relevant demographics, pain, and other psychological variables. Elucidating the function of hope in physical behavior among this population could present a new target for intervention.

## 2 Methods

### 2.1 Participants

The participants included 52 adults (17 males and 35 females). Participants were included if: they were between the ages of 55 and 85, community-dwelling men and women, had access to a smartphone, and answered yes to the following question: Have you experienced any musculoskeletal pain (pain affecting joints, bones, ligaments, tendons or muscles) in the past month? Individuals were excluded if: they had a history of cardiovascular issues such as uncontrolled blood pressure over 150/95 mmHg, heart failure, history of acute myocardial infarction, history of any systemic disease or physical condition (e.g., severe osteoarthritis, injury) that restricted normal daily activity, history of neurological disease (e.g., Parkinson’s Disease, multiple sclerosis, epilepsy), serious psychiatric illness (schizophrenia, bipolar disorder) or hospitalization within the preceding year for psychiatric illness, or a history of peripheral neuropathy. Participants were recruited in the greater Indianapolis area through newspaper, email, social media, or posted advertisements.

### 2.2 Procedure

Participants were asked to complete one study session that included the informed consent process, health history assessment, and completion of a series of questionnaires. As part of this process, participants were asked questions regarding the location and duration of pain. Prior to doing anything else related to the study, research staff explained the study and reviewed the informed consent verbally with each participant. Participants were given time to read through the informed consent and ask questions. All participants signed the Informed Consent Form. To determine eligibility, participants completed a health history questionnaire. Participants then completed a series of additional questionnaires, which are described below. Additionally, participants were given an accelerometer and instructions on how to wear it. They were instructed to wear it for 7 consecutive days following the study session. Participants were also given an accelerometer diary in which they recorded the start and finish times of wearing the accelerometer each day, as well as when they took the accelerometer off. This study was approved by the Indiana University Institutional Review Board and all participants provided written informed consent prior to participating in the study.

### 2.3 Outcome measures

#### 2.3.1 Objective physical activity with accelerometry

During the study session, participants were given an ActiGraph wGT3X-BT tri-axial accelerometer (ActiGraph^™^, Pensacola, Florida) to measure regular PA levels. Participants were instructed to wear the accelerometer on their right hip for seven consecutive days following the initial study session. The Actigraph wGT3X-BT is a small lightweight tri-axial accelerometer that is designed to detect tri-axial accelerations in the range of 0.05–2 G. Wear time validation was performed with the Choi parameters using the ActiLife 6 software, which classifies accelerometer wear and non-wear time (Choi et al., 2011). A valid accelerometer collection day was defined as wearing the accelerometer for ≥ 10 h/day. Data download, reduction, cleaning, and analysis were conducted using the ActiLife 6 software. The Sasaki et al. cut points using the vector magnitude were used to classify PA as moderate to vigorous (≥2,690 step count/min) (Sasaki et al., 2011). For each valid day (≥ 10 h of wear time), the time spent in moderate-vigorous PA (MVPA) and steps per day were calculated for each participant (Migueles et al., 2017). Participants were also provided with an accelerometer diary in which they were asked to record the start and finish times for each day, as well as the duration and reason for any period the accelerometer was removed.

#### 2.3.2 Physical activity scale for the elderly (PASE)

The PASE has a total of 18 items using a 4-point scale and yes/no questions (Washburn et al., 1993). The items capture seven dimensions of PA, encompassing work-related, household, and leisure activity. People with higher scores are more physically active. The PASE shows good validity as a measure of PA in older adults (Washburn et al., 1999).

#### 2.3.3 Adult hope scale (AHS)

AHS is a measure of trait hope in adults formed from agency and pathways thinking. The AHS includes 12 statements that are separated into two subscales, agency and pathways. The survey consists of four questions dedicated to agency (Hope-Agency), four questions dedicated to pathways (Hope-Pathways), and four filler items. Participants rated each item for statement accuracy on an 8-point scale (1 = definitely false; 8 = definitely true). An individual’s Total Hope Score is calculated by adding the eight items that comprise the subscales. The AHS has shown good convergent and divergent validity and represents trait hope (Snyder et al., 1991).

#### 2.3.4 Short-form health survey-36 (SF-36)

The SF-36 is a health survey that yields 8-scale scores (physical functioning, role limitations due to physical problems, bodily pain, vitality, general health perceptions, social functioning, role limitations due to emotional problems, and mental health). This tool was used to measure bodily pain in the current study. The survey responses are organized in a 3-to-5-point Likert Scale or binary response format, which is scored out of 100. Higher scores on the SF-36 indicate lower bodily pain and better functioning, while lower scores point to higher levels of pain and decreased functioning (Ware et al., 2000). The SF-36 has been found to be a reliable and valid tool for measuring general health in community-dwelling individuals (Brazier et al., 1992).

#### 2.3.5 Pain catastrophizing scale (PCS)

The PCS asks the respondents to reflect upon past painful experiences and to rate the degree to which they experienced negative thoughts or feelings about pain. Catastrophizing is a multidimensional construct composed of rumination, helplessness, and magnification (Sullivan et al., 1995). The questionnaire consists of four items examining rumination, five items related to helplessness, and three items associated with magnification rated on a 4-point scale (Ware et al., 2000). The PCS has been deemed a valid and reliable method for measuring pain catastrophizing in community-dwelling individuals with pain (Osman et al., 1997).

#### 2.3.6 Tampa scale of kinesiophobia-11 (TSK-11)

The TSK consists of 11 items and is rated on a 4-point scale. The TSK is used to measure the fear of movement with pain or re-injury (Kori et al., 1990). The TSK has been deemed a valid and reliable method for assessing fear of movement in both clinical and nonclinical populations (Goubert et al., 2004; Vlaeyen et al., 1995).

#### 2.3.7 Pain body map

Participants completed a validated pain body map (Scherrer et al., 2021). Males were given a male body map, while females were given a female body map. The maps presented two side-by-side posterior and anterior illustrations of the body with a line separating the right and left sides. The entire body map comprised of 74 body regions. Participants were told to shade in the regions on the map in which they had experienced pain in the last 7 days. The sum of all the shaded regions on the map equals the total number of body regions experiencing pain.

### 2.4 Statistical analysis

A power analysis using G Power 3.1.9.7 indicated that 52 participants were needed for predicting the change in R^2^ in a multiple linear regression model that included three covariates, with an estimated moderate effect size (f^2^ = 0.16), a power of 0.80, and alpha set at *p* = 0.05.

Descriptive statistics were calculated for all outcome measures. Shapiro–Wilk’s test of normality indicated that age, PCS, all Hope variables, and MVPA as well as steps derived from the accelerometer were not normally distributed; thus Mann–Whitney U tests were conducted to determine if theses variables differed by sex. Independent t-tests were conducted to determine whether the normally distributed variables differed by sex.

Bivariate Spearman’s Rho correlation analyses were conducted to determine the relationships of the PA variables with the Hope variables, age, bodily pain on the SF-36, TSK, and pain catastrophizing on the PCS. Hierarchical regressions were conducted to determine whether Hope predicted objective and subjective PA, after controlling for covariates (e.g., age, sex, bodily pain). For all regressions, age and sex were entered in step 1, SF-36 Bodily Pain subscale was entered in step 2, and the Hope variable was entered in step 3.

## 3 Results

### 3.1 Participant characteristics

Table 1 presents the descriptive statistics for participant demographics and all outcome measures separated by sex. Bodily pain on the SF-36 differed significantly between males and females. Males had higher scores (i.e., lower reported pain) on the SF-36 subscale compared to females. Back pain was reported by 75% of participants, with 44.2% of those experiencing it for more than 6 months. Neck pain was reported by 28% of participants, with 17.3% experiencing it for more than 6 months. Joint pain in the extremities (i.e., hands, arms, legs, or feet) was reported by 75% of participants, with 53.9% reporting pain duration of more than 6 months. In summary of the Pain Body Map results representing pain experienced in the last seven days, participants reported on average 7 ± 5.15 body regions with pain. The most common sites included the knee (57.7%), the lower back (57.7%), foot (36.5%), shoulder (34.6%), and hip (25%). Eight participants reported taking prescription medications for pain.

**Table 1:** Descriptive statistics for primary study measures in male and female participants.

Variable	Women (*n* = 35)	Men (*n* = 17)	*p*-value
Age, year	63.6 ± 7.0	64.9 ± 6.2	0.434
% aged 65 years or older	45.7%	41.2%	
BMI, kg/m^2^	28.9 ± 5.9	27.8 ± 4.2	0.822
Race, %			
African American	28.6%	11.8%	
Caucasian	68.6%	70.6%	
Hispanic	2.9%	5.9%	
Other	0.0%	11.8%	
% Taking prescription pain medications	20.0%	5.9%	
PASE score (0–793)	193.0 ± 83.1	225.4 ± 110.9	0.243
Steps per day	6,479 ± 2,241	7,730 ± 3,979	0.338
MVPA per day, minutes	35.6 ± 19.2	41.8 ± 32.5	0.946
SF-36 bodily pain score (0–100)	67.4 ± 14.1	78.1 ± 11.2	0.009*
Tampa scale of kinesiophobia (11–44)	18.5 ± 5.5	19.3 ± 4.8	0.619
Pain catastrophizing scale (0–52)	8.0 ± 11.0	6.5 ± 6.7	0.961
Total hope score (8–64)	56.1 ± 4.4	52.7 ± 9.4	0.338
Hope pathways score (4–32)	28.6 ± 2.5	25.8 ± 5.6	0.080
Hope agency score (4–32)	27.5 ± 2.9	26.9 ± 4.5	0.798

* = significant sex difference at the p = 0.05 level. Descriptives are presented as means and standard deviations or percentages. BMI = Body mass index; MVPA = moderate to vigorous physical activity; PASE = Physical Activity Scale for the Elderly.

### 3.2 Bivariate correlations

**Figure 1:**
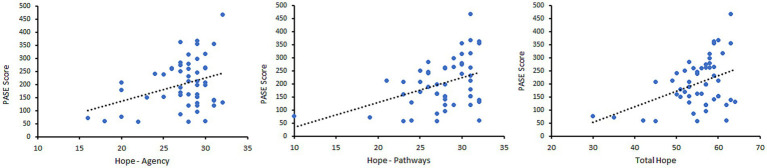
Scatterplots showing the relationship between PASE scores and Hope-Agency (left), Hope-Pathways (middle), and Total Hope score (right).

Table 2 presents the correlations (r values) between the PA variables and other outcome measures. Figure 1 provides scatterplots showing the relationship between PASE scores and the Hope variables. PA scores on the PASE positively correlated with Hope-Pathways (*p* = 0.017), the Total Hope Score (*p* = 0.022), steps on the accelerometer (*p* = 0.047), and bodily pain scores on the SF-36 BP (*p* = 0.004). Thus, greater self-reported PA was significantly related to greater hope and steps per day and lower bodily pain. Average steps per day was significantly correlated with bodily pain on the SF-36, with greater steps associated with lower pain (*p* = 0.004). Of note, scores on the PASE were not significantly correlated with steps and MVPA derived from the accelerometer.

**Table 2:** Bivariate correlation matrix between primary outcome measures.

	1	2	3	4	5	6	7	8	9	10
1. PASE	1.00									
2. MVPA	0.257	1.00								
3. Steps	0.280	0.785**	1.00							
4. Hope-Pathways	0.329*	0.075	0.108	1.00						
5. Hope-Agency	0.164	0.058	0.110	0.473**	1.00					
6. Total Hope	0.318*	0.059	0.109	0.868**	0.805**	1.00				
7. Bodily Pain (SF-36)	0.391**	0.238	0.393**	0.199	0.213	0.261	1.00			
8. TSK	−0.055	−0.072	−0.223	−0.153	−0.194	−0.235	−0.332*	1.00		
9. Pain Catastrophizing	−0.180	0.049	−0.039	−0.224	−0.150	−0.268	−0.440**	0.593**	1.00	
10. Age	−0.046	−0.154	−0.063	0.057	−0.038	−0.052	0.186	−0.056	0.016	1.00

* = *p* < 0.05; ** = *p* < 0.001. TSK = Tampa Scale of Kinesiophobia; PASE = Physical Activity Scale for the Elderly; MVPA = moderate to vigorous physical activity.

### 3.3 Hierarchical regressions

#### 3.3.1 Self-reported physical activity

Three separate regression models were conducted with the 3 separate Hope variables (i.e., Pathways score, Agency Score, Total Hope score) entered as the last predictor in each separate model. All three models were significant. After controlling for age, sex, and bodily pain, Hope-Pathways significantly predicted self-reported PA, accounting for 16.0% of the variance. Bodily pain was also a significant predictor, accounting for 14.3% of the variance. In the model with Hope-Agency as the final predictor, bodily pain but not Hope-Agency significantly predicted PA. In the model with the Total Hope Score as the final predictor, bodily pain approached significance and the Total Hope Score significantly predicted self-reported PA, accounting for 14.3 and 14.2% of the variance, respectively. A greater Total Hope Score and Hope-Pathways and lower bodily pain were associated with greater PA. See Table 3 for a summary of the significant hierarchical regression models for the PASE.

**Table 3:** Summary of significant hierarchical regression models for self-reported physical activity.

Step variables	ΔR^2^	Standardized *β*	*p* value for β	Model *p*-value
(A) Physical activity scale for the elderly (PASE)				
1. Age	0.034	−0.162	0.187	<0.001
Sex		−0.230	0.108	
2. Bodily pain	0.143	0.277	0.046	
3. Hope-pathways	0.160	0.446	0.002	
(B) Physical activity scale for the elderly (PASE)				
1. Age	0.034	−0.128	0.329	0.011
Sex		−0.085	0.546	
2. Bodily pain	0.143	0.324	0.030	
3. Hope-agency	0.064	0.265	0.053	
(C) Physical activity scale for the elderly (PASE)				
1. Age	0.034	−0.142	0.254	0.001
Sex		−0.186	0.184	
2. Bodily pain	0.143	0.264	0.063	
3. Total hope	0.142	0.414	0.003	

#### 3.3.2 Objective physical activity

Hierarchical regressions were conducted to predict average steps and MVPA per day as measured by the accelerometers. None of the regression models predicting MVPA were significant (*p* > 0.05). The regression models predicting steps per day were also not significant. However, the model predicting steps was significant (see Table 4) when only including steps 1 (age and sex) and 2 (bodily pain) in the regression model, with greater bodily pain predicting less steps per day. Bodily pain accounted for 10.6% of the variance in steps per day.

**Table 4:** Summary of significant hierarchical regression model for average steps per day.

Step variables	ΔR^2^	Standardized β	*p* value for β	Model *p*-value
1. Age	0.049	−0.145	0.293	0.046
Sex		−0.085	0.561	
2. Bodily pain	0.106	0.355	0.019	

## 4 Discussion

This study aimed to elucidate the relationship between trait hope and subjective and objective measures of PA in community-dwelling middle-aged and older adults with musculoskeletal pain. The data revealed several key findings. First, hope, particularly the pathways subscale, predicted self-reported PA, above and beyond bodily pain. Second, bodily pain, but not any of the hope outcomes, predicted steps per day measured via the accelerometer. These findings suggest that trait hope could be a key psychological factor facilitating overall PA levels in adults with musculoskeletal pain.

### 4.1 Trait hope’s prediction of self-reported physical activity

These results partially supported our hypothesis that hope, particularly agency, would predict self-reported PA. Higher levels of hope were associated with greater PASE scores even after controlling for bodily pain levels; however, this result was driven by the pathways versus agency subscale. This is in contrast to prior research showing that hope-agency predicts self-reported exercise frequency (Anderson and Feldman, 2020), perceived exercise goal attainment, and self-reported vigorous PA in younger adults (Blythe et al., 2025). The agency component is described as an individual’s capacity to utilize the strategized pathways (Yarcheski et al., 2004), while pathways refer to the different ways individuals can take to achieve their goals (Yarcheski et al., 2004). Perhaps hope pathways play a more important role in PA behavior in populations facing barriers to PA, such as persistent pain.

### 4.2 Trait hope’s prediction of objective physical activity

To our knowledge, this is the first study to objectively measure the impact of hope on PA. Contrary to our hypothesis, trait hope did not predict the accelerometer measures. However, bodily pain was a significant predictor of steps per day, suggesting older adults with more bodily pain accumulate less steps per day. This finding is in line with substantial evidence showing a negative relationship between PA behavior and musculoskeletal pain in older adults (Bruce et al., 2005; Hartvigsen and Christensen, 2007; Landmark et al., 2011).

### 4.3 Potential factors underlying the discrepant objective vs. subjective physical activity results

Several reasons could explain the contrasting self-report and objective PA results of the current study. First, previous research has shown a lack of concordance between objective and self-report measures of PA, with self-report measures often overestimating actual activity (Prince et al., 2008). For example, Hartvigsen and Christensen (2007) found a seven-minute increase in self-reported measures of MVPA compared to objective measurements (Liu et al., 2016). Second, these assessments are likely measuring different components of PA. Indeed, our correlation analyses indicated that the PASE scores were not significantly associated with steps and MVPA, even though the relationship trended in the expected direction. The accelerometers, which were worn on the hip, provide objective estimates of mostly ambulatory activity and may miss other activities such as swimming, lifting weights, and other activities primarily using the upper body (Ellis et al., 2014). On the other hand, the PASE attempts to capture all perceived activity, including dimensions covering work-related, household, and leisure activity that may encompass more than just ambulation (D’Amore et al., 2024). The PASE was designed to include activity thought to be more representative of typical activities performed by older adults, such as household tasks and gardening that might heavily involve the upper body. Third, it is possible that individuals higher in hope perceive more positive outcomes (Pleeging et al., 2021), such as completing more PA than they actually do.

### 4.4 Integrating results into hope theory and clinical relevance

The findings of this study indicate that pathways and bodily pain significantly influence self-reported PA behavior among community-dwelling adults with musculoskeletal pain. According to hope theory, the achievement of goals is partly dependent on the ‘routes’ individuals take to progress toward those goals (Yarcheski et al., 2004). It is possible that individuals who are more adept at generating pathways possess better pain-coping mechanisms, enabling them to engage in PA more effectively than those with a lesser ability to strategize. Alternatively, Snyder (Yarcheski et al., 2004) posits that “reciprocal temporal thinking” may elucidate pathway development by prompting individuals to take present actions that promote optimal wellness in the future. In this perspective, those with a greater capacity to create pathways might perceive a more negative future outcome if they choose not to exercise, motivating them to participate in PA to reduce the likelihood of further loss of functionality. These individuals might already possess the agency to use positive coping and rely on the generation of pathways to achieve their goals related to PA. Furthermore, Landmark et al. (2011) found that individuals exhibiting more positive traits, such as hope, tend to experience lower levels of pain catastrophizing and demonstrate better adaptability to painful stimuli. This could lead to greater agency and reliance on the ability to create pathways for predicting PA behavior.

Prior research suggests that psychological therapies, such as cognitive-behavioral therapy (CBT), can be successfully utilized in a multidisciplinary approach to address psychosocial factors linked to musculoskeletal pain (Hassett and Williams, 2011). By reframing maladaptive thoughts and behaviors, these therapies assist individuals in fostering greater engagement in health-promoting behaviors (Ehde et al., 2014). Results from a qualitative study examining the effectiveness and behavioral changes following a mind–body intervention, which included CBT, indicated that increased mind–body awareness and the establishment of realistic goals led to positive beliefs about exercise in a sample of adults with musculoskeletal pain (Misje et al., 2024). Similarly, another study found that combining physical exercise with CBT showed greater benefits in modifying exercise-related thoughts and behaviors by improving coping mechanisms in a sample of adults with musculoskeletal pain (Cheng and Cheng, 2019). Hope has also been studied as a target for intervention in the adjustment and management of chronic pain. Results from a study examining the influence of hope on pain tolerance and pain thresholds using a cold pressor task found that women experienced increased hope, and all participants experienced an increase in pain tolerance post-intervention (Berg et al., 2008). Thus, targeting hope to improve health behaviors, such as PA, and pain management in older adults could be a promising approach, but additional research is needed.

### 4.5 Limitations

This study has several methodological limitations. First, the cross-sectional design limits directionality conclusions regarding the relationship between PA and hope. Second, the inclusion criteria focused on general musculoskeletal pain instead of targeting a sample with specific musculoskeletal conditions. However, this approach strengthens the external validity of the study, as the sample likely represents the typical clinical pain experience for middle-aged to older adults. Indeed, research indicates that over half of older adults experience musculoskeletal pain in multiple body sites at once, with the knees, low back, and hips being the most common sites (Fejer and Ruhe, 2012). Participants in the current study reported experiencing pain in the past week on average at seven different body regions on the pain map, with the most common being the knee, low back, hip, and shoulder. Even with this variation in pain experiences within our sample, we still demonstrated a relationship between hope and self-reported physical activity. Given the reported location of pain in our sample, this study’s results most likely generalize to those with back and extremity joint pain. Future research should explore the hope-PA relationship within specific pain conditions, such as low back pain, fibromyalgia, and knee osteoarthritis. Further, the participants represented both acute and chronic phases of pain. The stage of their pain may potentially influence their responses to the self-report measures. Third, this study does not account for or measure state hope in predicting PA behavior within this sample, which limits the analysis to the effects of trait hope alone. Importantly, Anderson and Feldman (2020) found that state hope versus trait hope was a stronger predictor of exercise outcomes. Fourth, the sample size was relatively small, had a gender imbalance (67% females), and we did not assess the cognitive status of participants. However, no participants enrolled in this study presented with obvious cognitive deficits based on researcher-participant interaction during the study session. Fifth, a person’s experience of pain is multidimensional, with sensory, affective, and evaluative components. Our measure of pain (i.e., the Bodily Pain scale on the SF-36) did not evaluate these different components of pain. Whether certain dimensions of the pain experience pose a larger barrier to being physically active is unknown and could be an important avenue for future research. Finally, future research should also simultaneously assess other positive psychology constructs, such as self-efficacy and optimism, to deduce the most important psychological factors facilitating PA behavior in different populations. Self-efficacy is the belief that one can successfully engage in a behavior, while optimism refers to positive outcome expectations. Interestingly, two studies have found hope to be a stronger predicter of exercise or exercise goal attainment compared to optimism and self-efficacy (Anderson and Feldman, 2020; Blythe et al., 2025).

## 5 Conclusion

The extent of existing literature examining the role of hope in predicting PA is limited, and non-existent in pain populations. We provided the first evidence that trait hope influences self-reported PA levels above and beyond bodily pain in older adults with musculoskeletal pain. Older adults with low hope levels could be at a greater risk of sedentary behavior. Future research should consider studying this relationship using a longitudinal design, a more distinct sample, and adding measures of state hope. These methodological modifications will contribute to furthering our understanding of the role of hope in predicting PA behavior in older adults with musculoskeletal pain.

## Data Availability

The raw data supporting the conclusions of this article will be made available by the authors, without undue reservation.
